# Soft Gripper with Electro-Thermally Driven Artificial Fingers Made of Tri-Layer Polymers and a Dry Adhesive Surface

**DOI:** 10.3390/biomimetics7040167

**Published:** 2022-10-15

**Authors:** Xiangmeng Li, Qiangshengjie Shi, Huifen Wei, Xiaodong Zhao, Zhe Tong, Xijing Zhu

**Affiliations:** 1 Shanxi Provincial Key Laboratory of Advanced Manufacturing Technology, North University of China, Taiyuan 030051, China; 2 State Key Laboratory for Manufacturing Systems Engineering, Xi’an Jiaotong University, Xi’an 710049, China

**Keywords:** soft gripper, artificial finger, ecoflex–graphene composite, thermoplastic polyurethane elastomer (TPU), gecko-inspired dry adhesive, electro-thermal bending

## Abstract

Soft grippers have attracted great interest in the soft robotics research field. Due to their lack of deformability and control over compliance, it can be challenging for them to pick up objects that are too large or too small in size. In particular, compliant objects are vulnerable to the large grasping force. Therefore, it is crucial to be able to adjust the stiffness of the gripper materials. In this study, a soft gripper consisting of three artificial fingers is reported on. Each of the artificial fingers is made of a tri-layer polymer structure. An exterior layer, made of an ecoflex–graphene composite is embedded with electric wires as a heating source, by applying direct-current potential. The Joule heat not only allows for deformation of the exterior layer, but also transfers heat to the middle layer of the thermoplastic polyurethane (TPU) elastomer. As a result, the stiffness of the TPU layer can be adjusted using electro-thermal heating. Meanwhile, the third layer consists of a polydimethylsiloxane replica as a supporting layer with a gecko-inspired dry adhesive structure. By applying voltage through electric wires, the artificial fingers can bend and, thus, the soft gripper can hold the objects, with the help of the dry adhesive layer. Finally, objects like a shuttlecock, tennis ball and a glass beaker, can be picked up by the soft gripper. This research may provide an insight for the design and fabrication of soft robotic manipulators.

## 1. Introduction

Soft robotics is a widely used terminology to define mechanical manipulators made of deformable materials and structures [1]. Conventional rigid robots made with stiff materials, like steel or alloys, need to be manufactured using traditional machining tools. Soft robots are mostly made of polymers or polymeric composites with low Young’s modulus. These soft robotic manipulators can be fabricated by three-dimensional (3D) molding or 3D printing approaches in a cost-effective manner [2,3]. Unlike the rigid robots, soft robotic manipulators can be used to operate in unstructured scenarios, such as search and rescue missions [4,5,6].

Diverse actuation mechanisms can be used for soft robotic manipulators, including fluid, heat, electric and magnetic fields. Among the various actuation mechanisms for soft manipulators, actuation driven by hydraulic systems or pneumatic actuators are most commonly used. Hydraulic systems possess high power density and can generate large force and torque [7]. Meanwhile, pneumatic actuators show lower power but are more complex to control because of compressibility of gas. As a particular type of pneumatic actuator, jamming of granular media was proposed to change the stiffness of components [8,9,10,11]. Jamming-based grippers [8,9] can be used to pick up a wide range of objects without active feedback [10,11]. However, most fluidic-based soft manipulators are made of soft elastomers, which leads to low radial stiffness. Magnetically driven soft robotic manipulators have been developed in recent years for producing large actuation forces with magnetic fields [12]. However, their low power density is too limited to transfer energy to magnetic materials.

For electric field-based actuation, electrical energy can be converted to the soft materials, either by direct conversion into mechanical deformation or by indirect conversion through another form of energy. On the one hand, dielectric elastomer actuators (DEAs) are widely used for direct conversion to generate attractive force between the opposite electrodes to deform a soft elastomer dielectric [13,14,15,16,17,18]. DEAs can be prepared from low-cost materials and possess advantages of large strains and fast actuation speeds [19,20,21,22,23]. On the other hand, for indirect conversion, piezoelectric materials usually actuate materials of high modulus, leading to high stresses and fast actuation. However, one key drawback of piezoelectric actuation is low actuation strain [24]. Ionic electro-active polymer actuators (IEAPAs) work with movement of ions of electrolytic character in an electric field. IEAPAs can provide large bending movement at low voltage, but they have limitations, such as low force output and the requirement of strict encapsulation [25,26,27].

By transferring electrical energy to another form, many kinds of actuation mechanisms can be facilitated. A widely implemented indirect conversion from electrical energy is through Joule heating. Joule heat can allow for phase transformation in shape memory alloys (SMAs) [28] and liquid crystal elastomers (LCEs). LCEs present fully reversible thermal activation and achieve 60% contraction [29]. The electro-thermally induced phase transformation generates great stresses during actuation. The thermal energy can be converted into mechanical deformation due to differences of thermal expansion coefficients between the materials [30,31].

It is a simple strategy to employ Joule heat in certain materials to change the conditions of the component. For example, the phase change in polymers or polymeric composites changes the volume of the component [32], thus, enabling bending of soft actuators [33,34]. Miriyev et al. developed a flexible composite material to make high-strain artificial muscles with internal-doped ethanol [35], by electrical heating. Under the extreme volume change of the liquid–vapor phase transition, the artificial muscle expands and bends. It is of low voltage and cost effective for soft manipulators. Huan et al. proposed a type of soft grasper based on morphing jaws that increase contact area with clutching force [36]. In addition, biomimetic engineering has given rise to advancements in soft robotics. Shao et al. proposed gecko-inspired microstructures, which could achieve highly reversible adhesion to objects with van der Waals between the microstructures and objects [37,38,39,40]. Therefore, it is feasible to equip soft manipulators with dry-adhesive microstructures, to improve the performance of the soft manipulators. Particularly, the gripper can grasp objects without thermal heat dissipation in vacuum conditions; for instance, grasping manipulation in space exploration with high vacuum conditions, as well as in situations where lower acceleration is highly desired.

In this paper, we demonstrate a soft gripper with three artificial fingers. The artificial fingers consist of a tri-layer structure, including an upper layer, middle layer and a lower layer. Among the layers, the upper layer is made of silicone rubber, Ecoflex00-50, and a graphene composite and it plays the role of the initial driven layer. In addition, ethanol is embedded in the upper layer and plays a role as a liquid–vapor phase change material. During electrical heating, phase transition of the materials can occur and results in an extreme change in volume so that the artificial finger can deform. Meanwhile, the middle layer adopts a thermoplastic polyurethane (TPU) elastomer to alter the stiffness. The third layer consists of a polydimethylsiloxane (PDMS) film and a dry adhesive surface to improve the grasping performance. Moreover, a soft gripper can be obtained by assembling three artificial fingers and functionalizing with Joule heating. The design, fabrication and performance of the artificial finger is investigated.

## 2. Methods

### 2.1. Preparation of Actuating Layer for Artificial Finger

For the upper layer, platinum-catalyzed silicone rubber, Ecoflex00-50 (Smooth-On, Pennsylvania, USA), was adopted as the matrix material for the artificial finger. A mixture of part A and part B of Ecoflex00-50, in a mass ratio of 1:1, was prepared, and ethanol (Zhiyuan Chemicals, Tianjin, China) was added to the mixture with a volume ratio of 20% as a phase change material. A nickel-chromium alloy wire was embedded into the silicone rubber for electrical heating. The boiling point of ethanol allowed for rapid transformation from a liquid state to a vapor state, and the volume expanded rapidly, thereby driving the artificial muscle to bend. In addition, the Joule heat could not only bend the actuating itself, but also transfer heat to the adjacent layer. Reduced graphene oxide (rGO, XF Nano, Nanjing, China) was added to the ecoflex00-50 as a filler with a mass ratio of 3wt% and performed as a good thermal conductor to improve the thermal conductivity. During the experiment, the two components of ecoflex00-50 were mixed with rGO and ethanol by stirring, and the ethanol was in a liquid state in the mixture. The mixture of ecoflex00-50 and rGO was stirred thoroughly and poured into a finger mold with a coiled heating wire. Upon being kept still for 3 h, the mixture could be cured at room temperature, or it could be heated at a temperature under the boiling point of ethanol to enhance the curing. Then, the actuating layer of the artificial finger could be obtained after de-molding with full curing of the composites. We could observe that the ecoflex-rGO composite was solidified. As referred to in Aslan Miriyev’s report in Ref. [35], the ethanol would not interfere with the final curing state in the ecoflex-rGO composite as the driving layer.

### 2.2. Preparation of TPU Films with Variable Stiffness

For the middle layer, TPU films were prepared and the rheological properties were characterized. TPU powder (TPU C60, Elastollan TPU, BASF, Germany) with a melting point of 60 °C was selected as the material for preparing the variable stiffness layer. The powder was melted by hot pressing and kept at a temperature of 70 °C for 10 min, then cooled down to room temperature naturally. Upon peeling off, a TPU film with a thickness of about 1 mm was prepared and cut into a suitable size. In order to investigate the mechanical properties during heating of the TPU films, standard samples were prepared for testing the rheological properties. A dynamic thermomechanical analyzer (DMA850) was used to heat up the prepared TPU sample, and DMA Oscillatory TTS Ramp mode was employed. Moreover, a load was applied to measure the curves. By slowly heating up the TPU samples from 20 °C to 70 °C, the storage modulus and loss modulus could be obtained in respect to temperature. In addition, the variation of Young’s modulus and stiffness of a single TPU sample was investigated with the tensile apparatus while increasing the temperature.

### 2.3. Preparation of PDMS Thin Film with Mushroom-like Microstructures

For the lower layer, a PDMS replica was prepared by employing a molding approach from a template of gecko-inspired dry adhesive microstructures [40]. Briefly, photolithography was done to prepare mushroom-like microstructures. AZP4620 positive photoresist (AZ Electronic Materials USA Corp.) was used to fabricate sacrificed template, and a piece of square-shape glass was used as substrate for the sacrificed layer. The main steps included spin coating, pre-baking, mask alignment UV exposure, and development. Eventually, the template with a mushroom-like microstructure could be fabricated. After that, an amount of polydimethylsiloxane (PDMS, Dow Corning, Silgard 184) and curing agent, with a ratio of 10:1 in weight, were mixed by stirring and de-gassed in a vacuum prior to being poured onto the template. Then, PDMS was kept still in an oven at 60 °C for 1 h. Finally, a PDMS film with a dry adhesive mushroom-like microstructure was obtained by demolding from the sacrificial photoresist layer.

### 2.4. Assembly of Soft Gripper and Characterization

The three layers of polymeric structures were glued together with a thin layer of PDMS cured under ambient conditions to form a complete single artificial finger. By applying an appropriate DC voltage, the dynamic deformation of the finger could be observed. The process of bending of the artificial finger was recorded. During the test, a DC power supply with a voltage of 24.92 V was applied, and an output current of 1.085 A was observed. Then, three artificial fingers were assembled to make a soft gripper.

The morphology of the microstructure was characterized using a scanning electron microscope (FE-SEM, SU8000, Hitach, Japan) and a confocal microscope (LEXT4000, OLMPUS, Japan) with 50X objective lens.

### 2.5. Test of Dry Adhesive Films

To investigate the adhesion performance of the gecko-inspired dry adhesive microstructures, a series of PDMS samples were prepared, with mushroom diameters of 10 μm, 20 μm, 30 μm and 40 μm, respectively. An adhesion test of the dry adhesive films was conducted using a tensile force measurement machine (PT-1198GTD), with minimum precision of 2 mN. A clean glass slide, 5 mm × 5 mm, was adhered to a force-measuring probe, leading to a controllable contact area of 25 mm^2^. Four different diameter mushroom-shaped microstructure samples were tested for several preload forces from small to large. The relationship between the maximum adhesion forces and preload was obtained with a preload ranging from 0.2 to 1.5 N. The adhesion test was repeated three times for each sample.

## 3. Results and Discussion

### 3.1. Design of the Artificial Finger and Soft Gripper

**Figure 1:**
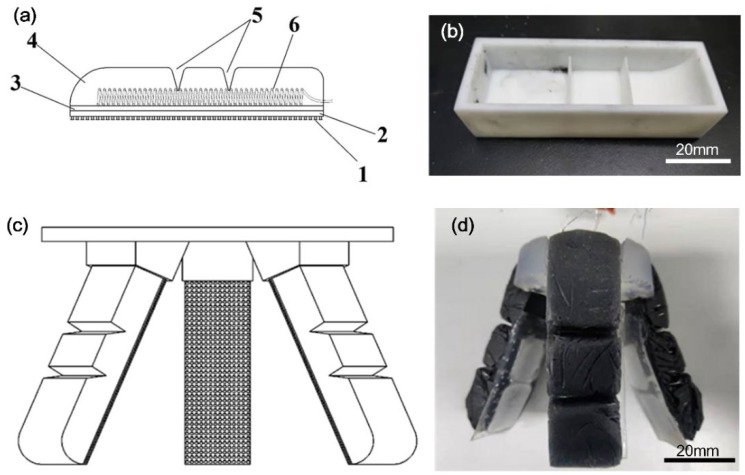
Design and fabrication of soft gripper. (**a**) Schematic model with number 1 to 6 indicating the dry adhesive microstructures (1), PDMS supporting layer (2), TPU layer (3), ecoflex–rGO composite layer (4), V-shape joints (5) and electric wires (6), respectively. (**b**) 3D printed mold for artificial finger. (**c**) Schematic illustration of assembly and (**d**) photo of the as-prepared soft gripper assembled from three artificial fingers.

Figure 1a depicts the distribution of the tri-layer structure of each artificial finger. As can be seen, the upper layer consisted of ecoflex–rGO composite, V-shape joints and embedded electrical wires, while the middle layer was made of a TPU layer and the lower layer was made of a PDMS supporting layer and the dry adhesive microstructures. Figure 1b shows the 3D printed mold for preparing the upper layer of the artificial finger. The V-shape joint could be made by molding from the ridge structures, as shown in Figure 1b, and they play a role of joint regions, where bending motion can occur during actuation. By assembling the artificial fingers onto the mount set, as shown in Figure 1c, a soft gripper could be obtained. The finally assembled soft gripper is demonstrated in Figure 1d. In order to keep it as flexible as possible to bend and fit the contact surface of grasped objects, we made a layer of PDMS, 1 mm in thickness, controlled by a glass slide. As shown in Figure 1d, the white squares indicate the TPU layer, and the mushroom-like dry adhesive structures are the transparent region with the supporting PDMS film. Meanwhile, the TPU layer played a role in changing the stiffness, thus, bending in response to Joule heating and recovering with natural cooling down.

### 3.2. Thermodynamic Performance Test of TPU Film

**Figure 2:**
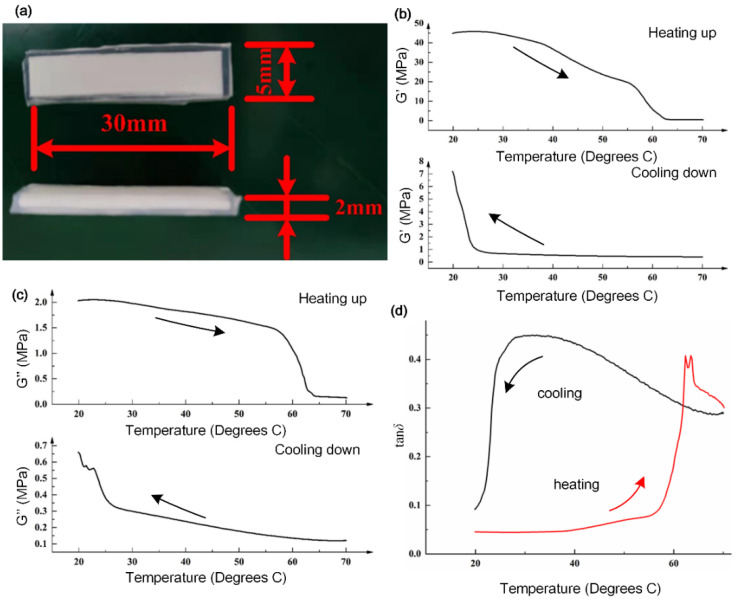
Sample of TPU and DMA test. (**a**) TPU samples encapsulated in PDMS thin films for testing. (**b**) Storage modulus (G’) of the TPU samples with heating up and cooling down processes. (**c**) Loss modulus (G’’) of the TPU samples with heating up and cooling down. (**d**) Loss angle (δ) of the TPU sample for both heating and cooling processes.

It is essential to figure out the relationship between stiffness and temperature for the TPU film, as this affects the actuating efficiency. Figure 2 shows that the stiffness of the TPU material could be changed during heating up or cooling down. The storage modulus and loss modulus indicated the rheological behavior during temperature variation. By this means, the variation of stiffness and actuation of the artificial fingers was analyzed quantitatively. For a better test of the rheological properties, the TPU films were made into cuboid samples, as shown in Figure 2a, with a length of 30 mm, a width of 5 mm and a thickness of 2 mm, and encapsulated with PDMS. During the heating process, the TPU layer would not outflow to contaminate the equipment.

Figure 2b shows that the TPU sample exhibited a high stiffness at room temperature, and its storage modulus was 44.84 MPa at the initial temperature of 19.89 °C. At a temperature range from 24.18 °C to 70.15 °C, the storage modulus of the sample gradually decreased. When the temperature reached about 63.85 °C, the sample started to melt, and the storage modulus at this moment tended to decrease slowly, until it reached a minimum value of 0.42 MPa. As soon as the heating was stopped, the liquid nitrogen cooling started and the storage modulus of the sample slowly rose with decreasing temperature, increased significantly after 24.42 °C, and, finally, reached 7.20 MPa at room temperature.

Figure 2c shows that the loss modulus of the TPU sample was 2.03 MPa at room temperature. The loss modulus of the TPU sample started to decrease from 2.05 MPa at a temperature of 22.45 °C, until it reached 0.16 MPa at 64.06 °C. The loss modulus of the sample decreased and tended to be stable, and slowly decreased to a plateau with a minimum value of 0.13 MPa. As the temperature decreased, the loss modulus gradually increased. When it reached room temperature, there was a small fluctuation and an upward trend. At this moment, the loss modulus was 0.66 MPa. The loss angle denotes the tangent of the phase difference angle between the strain and the stress cycle of the viscoelastic material, described as the ratio of loss modulus to storage modulus:

(1) $$\tan\delta =\frac{G^{\prime \prime }}{G^{\prime }}$$

where *δ* is the loss angle, *G′* is the storage modulus, and *G’’* is the loss modulus. According to the relationship between the storage modulus and the loss modulus of the sample, the relationship between the loss angle and temperature of the TPU was drawn.

**Figure 3:**
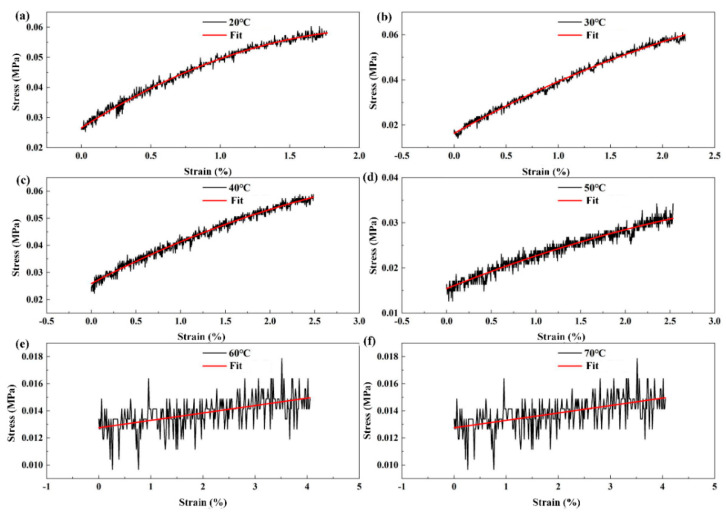
Stiffness variation of a single TPU sample with increasing temperature. (**a**–**f**) show stress–strain curves at 20 °C, 30 °C, 40 °C, 50 °C, 60 °C and 70 °C, respectively. The fitting curves indicate the nearly linear relationship between stresses and strains of in the TPU sample at different temperatures.

Figure 3 demonstrates the stress to strain curves as temperature increased. In order to compare the properties, only one TPU sample was tested in different temperature conditions. The slopes of the curves indicate the Young’s modulus, calculated as 2 MPa, 1.8 MPa, 1.2 MPa, 0.6 MPa, 0.05 MPa and 0.048 MPa, respectively. These comparative results directly indicated the variation of stiffness of the TPU film as a function of temperature.

### 3.3. Morphology and Test of Dry Adhesive Layer

**Figure 4:**
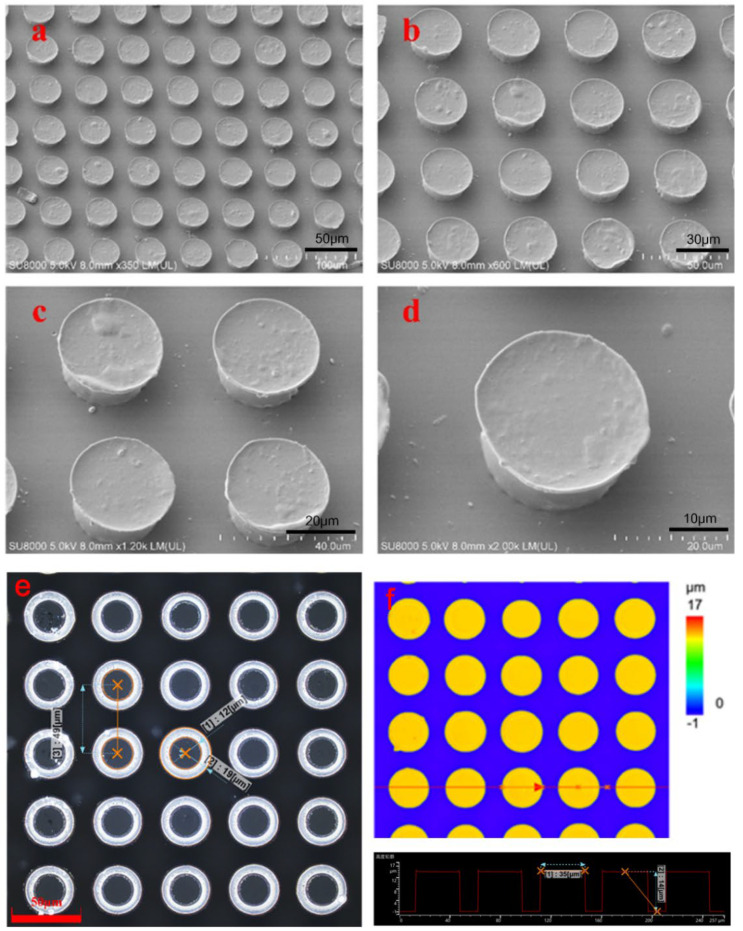
Morphological characterization of the dry adhesive microstructures. (**a**–**d**) Different magnified SEM images of the 20 μm sample. (**e**) Confocal microscope image showing the diameter of the head and pillar, and the distance between adjacent mushroom structures. (**f**) Confocal microscope image indicating the profile and height of the mushroom structures.

Figure 4a–d shows the SEM images of the mushroom-shaped microstructure of the dry adhesive surface. The mushroom-shaped microstructure film sample had a cap diameter of 30 μm. Each of the samples had a thickness of 1 mm and a center-to-center distance of 1.5 times the diameter. The morphology was observed by means of a confocal microscope. A relatively regular arrangement and smooth surfaces was found. There was no obvious structural damage to the structure. Figure 4e shows that the radius of the pillars was about 12 μm, which was the same as the aperture of the mask used. Meanwhile, the radius of the cap diameter was 19 μm, which was 58.3% higher than the original size, and the plane area increased by 150.69%. The measured center-to-center distance was 49 μm. The profile of the mushroom-shaped microstructures in the same straight line were observed. As shown in Figure 4f, the maximum width was 35 μm, and the height of the pillar structure was 14 μm. 

**Figure 5:**
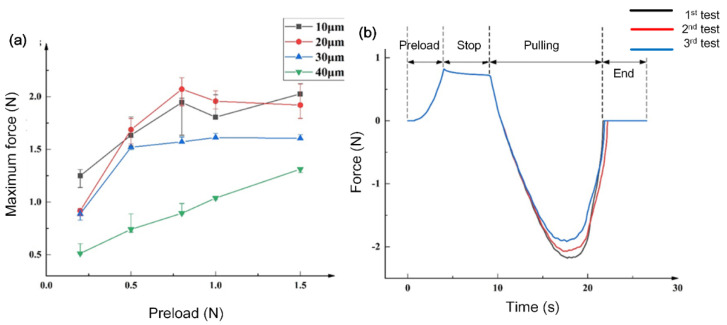
Dry adhesion test of the gecko-inspired dry adhesive surfaces. (**a**) Relationship curves between the maximum adhesive forces and the preload in comparing mushroom-like structures of 10, 20, 30 and 40 μm diameter. (**b**) Dry adhesive test of gecko-inspired structures with diameter of 20 μm, indicating the whole testing procedure, including preload and pulling process.

Figure 5 depicts the measurement results of dry adhesive forces. With increase in the preload force, the maximum adhesion forces of the other three samples with different diameters showed a trend of increasing first and then reaching a plateau (Figure 5a). The maximum adhesion force of the sample was found for a microstructure diameter of 20 μm when the preload force was 0.8 N. The fluctuation in the test process was related to the actual manipulation during the adhesion test.

According to the relationship between the preload force and the maximum adhesion force, we selected the sample with a diameter of 20 μm to test the relationship between the sample’s adhesion force and time under a preload force of 0.8 N. Figure 5b shows that the preload was gradually increased to the set value of 0.8 N. In the second stage of the curve, the tension meter retained a force of about 0.8 N, and increased with time. The third stage of the curve was the loading process. Upon reaching the maximum adhesion force, it decreased to zero until the force measurement probe was completely separated from the sample. The trend of the three repeated experiments was the same. The maximum adhesion force achieved by the effective contact area of 25 mm^2^ was 2.18 N, 2.071 N and 1.914 N as the number of experiments increased. The microstructure of the sample surface showed some wear on the head of the mushroom structure. The slight reduction of maximum adhesive force could have been due to the wear of the mushroom-like structure.

Herein, we compared four kinds of mushroom-like structures with varied diameters, i.e., 10, 20, 30, 40 μm, respectively. From the results of the adhesive test, we found that the maximum adhesion force was observed for the 20 μm structure. Since the morphology of the gecko-foot structure is a rather complicated hierarchical structure, we could only mimic the gecko-effect for achieving dry adhesion to some extent.

In this study, we did not take into account the PDMS surface without microstructure or with a smooth layer, because we only used the dry adhesive surface to enhance the grasping performance. Nevertheless, it was understood from a report by Shao et al. [38,39] that the dry adhesive structures could perform better in gripping objects, with the better morphology of the mushroom-like structure. Without the PDMS surface, the bended artificial fingers could still grasp the objects. However, the dry adhesive could facilitate the grasping performance by adding dry adhesion and direct contact with the surface of the objects. In addition, the Joule heat did not threaten the interfaces among the three layers, because a small amount of the same PDMS material was adopted as glue in between the layers, and could retain the gluing property even at 200 °C.

### 3.4. Performance of Single Artificial Finger

**Figure 6:**
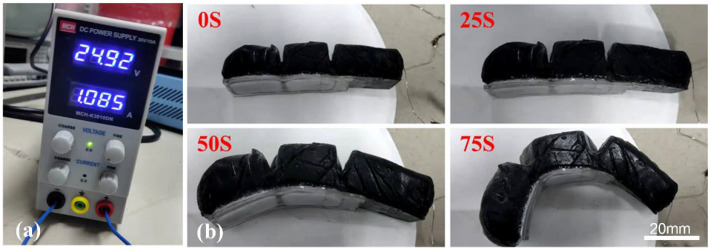
Electro–thermal driving test of the artificial finger. (**a**) DC power indicating the applied voltage and current for heating the artificial finger. (**b**) Captured photos recorded during the bending process of artificial finger in 75 s.

Figure 6 shows the process of driving an artificial finger. During the application of voltage, a picture was captured every 25 s. In the initial state, the finger was placed on the smooth surface, and the Joule heat began to accumulate. After 25 s, the finger expanded a little, but the change was still not obvious because the TPU layer remained highly stiff without enough heat. After 50 s, the finger had expanded greatly and begun to bend downward, so that the heat was transferred from inside to neighboring layers. After 75 s, the finger was fully expanded and bent to its maximum extent, due to enough heat having been generated inside the driving layer. As a result, the artificial finger deformed greatly with the help of the V-shape joint regions. The middle layer of TPU began to melt with enough heat. Thus, the lower stiffness could be adapted to various shapes of objects. The mixing of rGO in the Ecoflex00-50 made it easier to transfer heat to all parts of the artificial finger in a faster manner, which accelerated the entire expansion and bending processes. During electro–thermal actuation, the Joule heat softened the TPU layer, and the bending mode could be locked. By controlling the heating process, the TPU layer could retain the mechanical properties for a while during grasping of objects. Upon switching off the electrical power, the driving layer of ecoflex–rGO started to recover its shape, while the TPU layer was still soft enough to enable bending. Then, the TPU layer became stiff again until it cooled down to room temperature, which could be validated from the DMA results.

It took 1 to 2 min to actuate the artificial finger and 2 to 3 min to recover the shape because cooling took time. Indeed, it was not so fast either to drive or release the objects upon cooling down as the heating efficiency was not sufficiently good. Some measures could be taken, such as increasing the electrical power, to enhance the heating efficiency. In addition, we could use a water pump to facilitate the cooling by embedding tubes in the artificial finger.

### 3.5. Grasping Test of Soft Gripper

A regular triangle arrangement was adopted to mount the three artificial fingers to the base and form the soft gripper. In addition, the electrical wires were arranged in parallel to the three fingers. The advantage of parallel connection was that the overall total resistance could be reduced and the output voltage was reduced, thus reducing the energy consumption.

**Figure 7:**
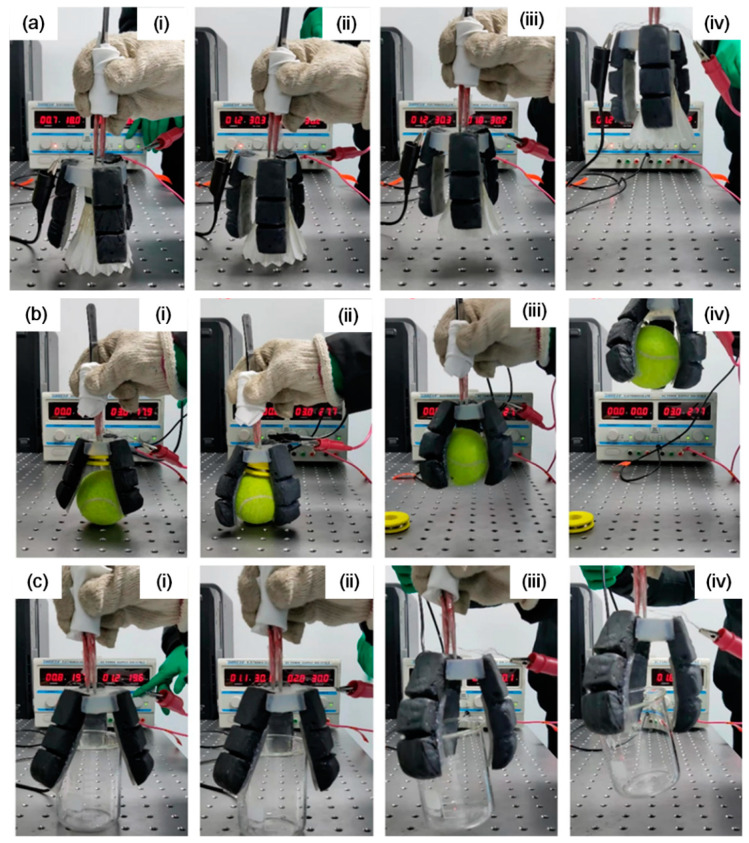
Demonstration of grasping objects (**a**) shuttlecock, (**b**) tennis ball and (**c**) glass beaker with the as-prepared soft gripper. (**i**–**iv**) indicate the initial state, bending starting and touching the objects, holding the objects and lifting the objects, respectively.

The behavior of the soft gripper in grasping objects was observed by applying DC voltage. Figure 7 demonstrates three different objects with the as-prepared soft gripper, including a shuttlecock with a mass of 4.7 g (Figure 7a), a tennis ball with a mass of 51 g (Figure 7b), and a 150 mL glass beaker with a mass of 68.7 g (Figure 7c). The photos were captured during the grasping processes. The soft gripper started to work as soon as the power was on. At the first stage, each artificial finger took time to accumulate heat, and there was no significant change in the fingers (i). With continued electrothermal heating, the soft gripper gained sufficient heat, and the fingers began to bend and touched the surface of the object (ii). Then, the fingers started to expand and bend significantly to grasp the object. Thus, the soft gripper held the objects firmly at about 15 mm from the table (iii). Later, the soft gripper kept rising to a height of about 40 mm, and at this moment, the objects could be held without falling down, owing to sufficient grasping forces (iv). Meanwhile, the soft gripper was compliant and not harmful to the grasped objects. This process could last for a while, until the DC power supply was switched off and cooling down commenced. The soft gripper could grasp both regular and irregular objects with smooth or rough surfaces. The demonstration of the soft gripper grasping objects verified the feasibility of the design and fabrication of the soft gripper. To test the performance of the soft gripper with three artificial fingers, the DC voltage was 27.7 V and total current for the three artificial fingers was 3.0 A. Therefore, the total power consumption to drive the three fingers was about 83 W (each finger using about 28 W). A crucial drawback to be addressed is that it may take time to heat up the artificial finger, which is, perhaps, not acceptable in practical soft robotic manipulator application. However, it is possible to shorten the time for the bending motion by supplying sufficient voltage and current to increase the Joule heating power to facilitate bending more efficiently. For instance, the heat transfer efficiency could be enhanced by adding more graphene. Nickel-chromium (Ni–Cr) alloy wires, which have higher thermal efficiency, could be adopted and the spatial configuration of electric wires could be modified to U-shape coils, thus, increasing the contact area and improving the heating efficiency. As for the cooling recovery procedure, we could design proper structures to reduce the heat to mechanical energy loss. Furthermore, we could finely design embedded water tubes in the artificial fingers to help cool down the fingers faster.

## 4. Conclusions

In summary, a soft gripper was presented with three artificial fingers made with tri-layer polymeric structures. The three functional layers were prepared by molding and pressing, and their mechanical or rheological properties were investigated. The actuation layer was an ecoflex–rGO composite with embedded electric wire, and it could generate heat and allow for liquid–vapor phase transition of the actuation layer. This layer transferred heat to the other layers so that the artificial fingers could bend around the V-shape joints. The volume could expand and drive the artificial finger to bend. The middle TPU layer showed effective variable stiffness, due to Joule heating, and it could be adapted to different shapes of objects. In addition, the dry adhesive film on the PDMS supporting layer revealed good adhesion in the test, which could help in grasping objects with various irregular shapes and surface roughness. By assembling three artificial fingers, a soft gripper was formed. The performance of grasping objects was demonstrated, showing that the prepared soft gripper could hold the objects by applying relatively low voltage. The soft gripper with a tri-layer composite structure has advantages with electrothermally actuated ecoflex–rGO composite as the driving layer, the TPU layer as a stiffness variable layer and the dry adhesive surfaces to enhance grasping. In particular, with the help of the softened TPU layer, the dry adhesive, with mushroom shaped microstructures might be more compliant to adapt to different shapes and roughness of objects. The limitation of the presented soft gripper was due to issues with the thermal transfer efficiency and it might take too long to cool down the artificial fingers to release the grasped objects. In future work, we can take some measures, such as adding higher power to enhance the heating efficiency and, at the same time, using a water pump to facilitate the cooling of the artificial fingers.

## Data Availability

Not applicable.
